# Impact of GnRH antagonist pretreatment on oocyte yield after ovarian stimulation: A retrospective analysis

**DOI:** 10.1371/journal.pone.0308666

**Published:** 2024-10-07

**Authors:** Federica Di Guardo, Sylvie De Rijdt, Annalisa Racca, Panagiotis Drakopoulos, Shari Mackens, Laurence Strypstein, Herman Tournaye, Michel De Vos, Christophe Blockeel

**Affiliations:** 1 Centre for Reproductive Medicine, Universitair Ziekenhuis Brussel, Vrije Universiteit Brussel, Brussels, Belgium; 2 Department of General Surgery and Medical Surgical Specialties, University of Catania, Catania, Italy; 3 Centre of Reproductive Medicine, Instituto Bernabeu Venezia, Martellago, Venezia, Italy; 4 Institute of life, IVF clinic, Athens, Greece; 5 School of Medicine, European University Cyprus, Engomi, Nicosia, Cyprus; 6 Department of Obstetrics, Gynecology, Perinatology and Reproduction, Institute of Professional Education, Sechenov First Moscow State Medical University of the Ministry of Health of the Russian Federation, Moscow, Russia; Kasr Alainy Medical School, Cairo University, EGYPT

## Abstract

The study investigates whether a 3-day pretreatment course with a GnRH antagonist in the early follicular phase has an impact on the number of retrieved COCs in a GnRH antagonist stimulation protocol. This is a retrospective single center crossover study involving women who did not conceive after one GnRH antagonist stimulation cycle (“standard cycle”) and proceeded with another GnRH antagonist stimulation cycle preceded by early administration of GnRH antagonist for 3 days (“pretreatment cycle”) with fresh embryo transfer or frozen embryo transfer. 430 patients undergoing 860 cycles were included. The mean female age was 34.4 ± 4.8 years. Indications for fertility treatment included unexplained infertility (34.3%), male-factor infertility (33.3%), age (16.9%), PCOS (8.2%), tubal (4.7) and endometriosis (2.6%). All cycles were divided into two groups: group 1 (standard, 430 cycles) and group 2 (pretreatment, 430 cycles). The mean duration of stimulation was similar in both groups (10.3 vs 10.3 days, p = 0.28). The starting dose of gonadotropin (234.9 vs 196.8 IU, p<0.001), total amount of gonadotropin used (2419 vs 2020 IU, p<0.001), the total number of retrieved COCs (10 vs 7.8 p<0.001) and the number of mature oocytes (8 vs 5.8 p<0.001) were significantly higher in group 2 than in group 1. The Generalized estimating equation (GEE) regression analysis showed that the pretreatment strategy had a significant positive effect on the number of COCs (coefficient 2.4, p <0.001 after adjusting for known confounders (age, indication, stimulation dose, type, and duration of stimulation). In conclusion, A 3-day course of GnRH antagonist pretreatment increases the number of COCs obtained after ovarian stimulation.

## Introduction

The use of gonadotropin-releasing hormone (GnRH) antagonists has been progressively increased in Assisted Reproductive Technique (ART) clinics worldwide, GnRH antagonists act by suppressing immediately and irreversibly the gonadotropin secretion, which results in a shorter duration of treatment with less patient distress [1–3]. Moreover, the use of GnRH antagonist protocol is associated with lower risk of hospital admission due to ovarian hyperstimulation syndrome (OHSS) [4]. On the other hand, several ART centers use the GnRH agonist protocol as first line option due to several reasons. First, the GnRH antagonist protocol has been associated with asynchrony antral follicle growth under certain condition [5]; second, the start of ovarian stimulation in a GnRH antagonist protocol relies on the occurrence of spontaneous menses [6–8] whereas the GnRH agonist protocol is more flexible allowing a more controlled scheduling of oocyte retrievals which means also the reduction or even the avoidance of oocyte retrievals during the weekend. In the clinical practice, pretreatment with Oral Contraceptive Pill (OCP) is used in antagonist protocols to obtain a more flexible scheduling of the start of ovarian stimulation. However, this practice is associated a decrease of ongoing pregnancy rate (OPR) [9, 10] as well as fresh and cumulative live birth rates (LBRs) [11]. Furthermore, an increase of duration of ovarian stimulation with higher gonadotropin consumption has been reported [12].

In addition, higher serum gonadotropin concentrations as well as higher E2 concentration are found at the onset of ovarium stimulation in GnRH antagonist protocol when compared with a pituitary down regulation protocol. As a result, the unsuppressed FSH level at the start of a GnRH antagonist cycles allows the initial growth of a few leading follicles before the addition of exogenous recombinant FSH (rFSH) [1, 13, 14]. Menstrual administration of an antagonist before starting ovarian stimulation might reduce size and improve homogeneity of antral follicles [5]. It has already been shown that elevated progesterone at the onset of ART cycles, and reduced fertility outcome, can be solved by the administration of GnRH antagonists for 3 consecutive days before the start of Ovarian Stimulation (OS) [15].

Furthermore, a pilot study conducted in women under 36 years old, found that GnRH antagonist pretreatment during 3 consecutive days before the initiation of ovarian stimulation had a trend towards a higher number of retrieved cumulus-oocyte complexes (COCs) with improved pregnancy outcome [16]. Using a similar protocol, improved maturation and fertilization rates of retrieved oocytes was showed [17]. The current study aims to investigate whether a 3-day pretreatment course with a GnRH antagonist in the early follicular phase may increase the number of oocytes retrieved in a GnRH antagonist stimulation protocol using a large data set.

## Material and methods

### Study design

This was a retrospective, single-centre cohort study (crossover, match–control design) at a tertiary referral university hospital including all consecutive women undergoing ovarian stimulation for In Vitro Fertilisation/ Intracytoplasmic Sperm Injection (IVF/ICSI) at Brussels IVF, the University Hospital of Brussels in Belgium from January 2011 to December 2020. The study was approved by the institutional Review Board of Universitair Ziekenhuis Brussel (approval B.U.N. 143201838385).

### Study population

Eligible patients were those who did not get pregnant after one standard GnRH antagonist stimulation cycle (“standard cycle”) and proceeded with one GnRH antagonist stimulation cycle preceded by early administration of GnRH antagonist for 3 days (“pretreatment cycle”) with fresh embryo transfer or frozen embryo transfer. All women may have used the same or a lower initial dose of gonadotropins in their first IVF cycle (standard cycle), both cycles needed to be performed in a time interval of <12 months.

The age of included patients ranged from 20 to 44 years. Patients were excluded from the study if they had planned to undergo ovarian stimulation for preimplantation genetic diagnosis or screening, oocyte donation, social or medical egg freezing and in vitro maturation (IVM) of oocytes. All women that had basal progesterone levels >1.5ng/ml, were deemed non-eligible. All cycles were divided into two groups: group 1 (standard cycles) and group 2 (pretreatment cycles).

### Treatment protocol

In standard cycles ovarian stimulation was started on day 2 or 3 of the menstrual cycle with daily injections of gonadotrophins, followed by a daily dose of 0.25 mg of GnRH antagonist in a fixed protocol, starting 6 days later. In pretreatment cycles patients started antagonist pretreatment on day 2 or 3 of the menstrual cycle for 3 days onwards. The day after finishing the pretreatment (day 5 or 6 of menstrual cycle) they started with daily injections of gonadotrophins, followed by a daily dose of 0.25 mg of GnRH antagonist in a fixed protocol, starting on the sixth day of stimulation.

Gonadotropins used were rFSH Gonal-F®, Merck Pharmaceuticals, Darmstadt, Germany; Ovaleap®, Theramex, Ireland Limited; Puregon®, Organon, Whitehouse Station, NJ, USA; or highly purified HMG (hpHMG) Menopur®, Ferring Pharmaceuticals, St. Prex, Switzerland. Cycle monitoring involved serum assessments of E2, P, FSH, LH, and serial transvaginal ultrasound examinations [18]. Ovulation was induced by administering hCG upon the observation of three follicles with a diameter of 17 mm [19]. Oocyte retrieval took place 36 hours thereafter. Collected oocytes were inseminated either via conventional IVF, ICSI or via IVF/ICSI. Embryos were cultured up to Day 3 or Day 5 following oocyte retrieval and the embryo transfer (ET) was performed under ultrasound guidance. Luteal phase support consisted in vaginal progesterone tablets of 200 mg three times daily, administered from the day after oocyte retrieval onwards until 7 weeks of pregnancy [20, 21]. In case of frozen ET of embryos obtained from the same cycle, hormonal replacement therapy (HRT), natural cycle (NC) and NC with triggered ovulation protocols were used to prepare the endometrium.

### Main outcome measures

The primary outcome parameter was the total number of retrieved COCs after ovarian stimulation. The secondary outcomes were consumption (IU) of gonadotrophins and duration (days) of ovarian stimulation.

### Statistical analysis

Continuous data are presented as mean ± standard deviation (SD) and median with interquartile range (IQR). Categorical data are described by number of cases, including the numerator and denominator, and percentages. Differences in continuous variables (including the primary endpoint: total number of retrieved COCs after ovarian stimulation) between patients’ 2nd IVF cycle (with GnRH antagonist pretreatment) and their preceding cycle were calculated via dependent-sample t-tests or Wilcoxon signed-rank tests, as appropriate. Categorical variables were analyzed via Mc Nemar test, as appropriate. Continuous variables were analyzed by regression models with estimation by generalized estimating equations (GEE) to assess the effect of antagonist pretreatment in the number of oocytes and embryo utilization rate, after accounting for several confounders such as dose of gonadotropin used, type of gonadotropin used, age, cause of infertility and duration of ovarian stimulation. GEE was used to account for the within subject correlation in outcomes for repeated treatments. Results are presented with adjusted odds ratios (ORs) and 95% confidence intervals (CIs). All statistical tests used a two-tailed α of 0.05. Analyses were performed using STATA 13.0. A p-value <0.05 was considered as statistically significant.

## Results

### Baseline patient baseline characteristics in the general population

In total, 430 patients undergoing 860 cycles were included. The average female age was 34.4 ± 4.8 years. Indications for fertility treatment included unexplained infertility (34.3%), male-factor infertility (33.3%), age (16.9%), PCOS (8.2%), Tubal-factor infertility (4.7%) and endometriosis (2.6%). All cycles were divided into two groups: group 1 (standard, 430 cycles) and group 2 (pretreatment, 430 cycles). The average cohort AMH value was 2.61 ± 2.52. Basal progesterone (assessed on day 2 or 3 of the menstrual cycle) was significantly higher in group 2 (0.66 ± 0.72 vs 0.51 ± 0.3, p <0.005) (Table 1).

**Table 1:** Baseline patient characteristics.

Parameter	n 430
**Age**	
**(mean ± SD)**	34.4 ± 4.8
**median (IQR)**	35 (31–38)
**AMH**	
**(mean ± SD)**	2.61 ± 2.52
**median (IQR)**	2.04 (1.01–3.43)
**Indication n (%)**	
**Male factor**	143 (33.3)
**Endometriosis**	11 (2.6)
**Age/ovarian insufficiency**	73 (16.9)
**Idiopathic**	148 (34.3)
**PCOS**	35 (8.2)
**Tubal**	20 (4.7)

### Stimulation characteristics in the two groups: Standard treatment and antagonist pre-treatment

Prior-triggering hormonal assessment revealed that E2, P, LH and FSH levels were significantly higher in Group 2 than in Group 1 (2289.7 ± 1355.6 vs 1628.4 ± 971.3, p<0.001; 1.02 ± 0.65 vs 0.88 ± 0.53, p<0.001; 3.9 ± 4.65 vs 2.5 ± 3.17, p<0.001; 18.08 ± 7.2 vs 15.8 ± 6.9, p<0.001, respectively) (Table 2).The mean duration of stimulation was similar in both groups (10.3 ± 1.6 vs 10.3 ± 2.2; p = 0.28) (Table 3). The starting dose of gonadotropin and the total amount of gonadotropins used were significantly higher in group 2 than in group 1 (234 ± 60.9 vs 196.7 ± 54.4 p<0.001; 2419 ± 758.4 vs 2020 ± 674.9, p<0.001) (Table 3). In both groups, rFSH, was more used than hMG [389/531(73.3) vs 142/531(26.7); 284/531 (53.5) vs 247/53 (46.5), p<0.001] (Table 3).

**Table 2:** Basal and prior-triggering hormonal assessment in the two groups: Standard treatment and antagonist pre-treatment.

Hormonal assessment	Standard treatment (430)	Antagonist pre-treatment (430)	P-value
	Basal hormonal assessment		
**E2 (ng/ml)** **(mean ± SD)** **median (IQR)**	39.8 ± 24.1 38 (27–50)	42.5 ± 19.9 39 (28–56)	0.006
**P (ng/ml)** **(mean ± SD)** **median (IQR)**	0.51 ± 0.3 0.45 (0.29–0.7)	0.66 ± 0.72 0.47 (0.3–0.8)	<0.005
**LH (IU/L)** **(mean ± SD)** **median (IQR)**	6.03 ± 2.71 5.75 (4.4–7.4)	6.18 ± 2.77 5.8 (4.2–7.7)	0.41
**FSH (IU/L)** **(mean ± SD)** **median (IQR)**	7.84 ± 2.97 7.6 (6–9.4)	7.68 ± 3 7.2 (5.8–8.9)	0.18
	Hormonal assessment at trigger		
**E2 (ng/ml)** **(mean ± SD)** **median (IQR)**	1628.4 ± 971.3 1461 (990–2094)	2289.7 ± 1355.6 1977.5 (1337–2972)	<0.001
**P (ng/ml)** **(mean ± SD)** **median (IQR)**	0.88 ± 0.53 0.8 (0.5–1.12)	1.02 ± 0.65 0.89 (0.6–1.31)	<0.001
**LH (IU/L)** **(mean ± SD)** **median (IQR)**	2.5 ± 3.17 1.6 (0.8–3.1)	3.9 ± 4.65 2.55 (1.2–4.69)	<0.001
**FSH (IU/L)** **(mean ± SD)** **median (IQR)**	15.8 ± 6.9 14.7 (10.8–20)	18.08 ± 7.2 17 (12.6–22.9)	<0.001

**Table 3:** Stimulation characteristics in the two groups: Standard treatment and antagonist pre-treatment.

	Standard treatment (430)	Antagonist pre-treatment (430)	P-value
**Gonadotrophin Starting Dose (IU) (mean ± SD)** **median (IQR)**	196.7 ± 54.4 200 (150–225)	234 ± 60.9 225 (200–300)	<0.001
**Total Gonadotrophin consumption (IU)** **(mean ± SD)** **median (IQR)**	2020 ± 674.9 2000 (1500–2400)	2419 ± 758.4 2250 (1800–3000)	<0.001
**Stimulation Length (days)** **(mean ± SD)** **median (IQR)**	10.3 ± 1.6 10 (9–11)	10.3 ± 2.2 10 (9–11)	0.28
**Type of gonadotropins *n* (%)** **rFSH** **hMG**	389/531(73.3) 142/531(26.7)	284/531 (53.5) 247/53 (46.5)	<0.001

### Stimulation and cycle outcomes in the two groups: Standard treatment and antagonist pre-treatment

The total number of obtained COCs and the number of mature oocytes were significantly higher in group 2 than in group 1 (10 ± 6.6 vs 7.8 ± 5.5, p<0.001; 8 ± 5 vs 5.8 ± 4, p<0.001, respectively; *difference in means 2*.*2 and 95% CI*: *from 1*.*6 to 2*.*9)*.

Fertilization rate, number of cryopreserved D3 Embryos, embryo utilization rate and the incidence and severity of OHSS were similar between the two groups [68 ± 27 vs 70 ± 25, p = 0.27; 0.3 ± 0.8 vs 0.47 ± 1.1, p = 0.08; 52 ± 36 vs 51 ± 33, p = 0.32; No OHSS: 531/531 (100) vs 529/531(99.6), Mild OHSS: 0/531 (0) vs1/531 (2), Moderate OHSS: 0/531 (0) vs 1/531 (2), p = 0.36]. The number of cryopreserved blastocysts was significantly higher in group 2 than in group 1 (1.09 ± 2.2 vs 0.28 ± 0.7, p <0.001) (Table 4).

**Table 4:** Stimulation and cycle outcomes in the two groups: Standard treatment and antagonist pre-treatment.

	Standard treatment (430)	Antagonist pre-treatment (430)	P-value
**COC** **(mean ± SD)** **Median (IQR)**	7.8 ± 5.5 7 (4–10)	10 ± 6.6 9 (6–14)	<0.001
**MII Oocyte** **(mean ± SD)** **Median (IQR)**	5.8 ± 4 5 (3–7)	8 ± 5 7 (4–11)	<0.001
**Fertilization rate** ^*a*^ **(mean ± SD)** **Median (IQR)**	68 ± 27 71 (50–100)	70 ± 25 75 (60–90)	0.27
**Cryo Embryos D3** **(mean ± SD)** **Median (IQR)**	0.3 ± 0.8 0 (0–0)	0.47 ± 1.1 0 (0–0)	0.08
**Cryo Embryos D5** **(mean ± SD)** **Median (IQR)**	0.28 ± 0.7 0 (0–0)	1.09 ± 2.2 0 (0–1)	<0.001
**Embryo** **utilization rate** ^*b*^ **(mean ± SD)** **Median (IQR)**	52 ± 36 50 (25–100)	51 ± 33 50 (25–75)	0.32
**Incidence and severity of OHSS** **n (%)**			
**No OHSS** **Mild OHSS** **Moderate OHSS**	531/531 (100) 0/531 (0) 0/531 (0)	529/531(99.6) 1/531 (2) 1/531 (2)	0.36

^a^calculated as number of oocytes fertilized divided by number of COC, multiplied by 100

^b^calculated as number the number of embryos utilized (transferred or cryopreserved) per number of 2PN zygotes

### Generalized estimating equation regression analysis

The generalized estimating equation (GEE) analysis showed that the pretreatment strategy had a significant positive effect on the number of COCs (coefficient 2.4, 95% C.I. 3.15 to 1.76, p <0.001), after adjusting for the confounders (age, indication of infertility, stimulation dose, type and duration of stimulation). On the other hand, the older age had a significant negative effect on the number of COCs (coefficient -.28, 95% C.I. -.38 to -.18, p<0.001) (Table 5).

**Table 5:** Generalized estimating equation (GEE) analysis. Outcome: number of COCs, predictors: Maternal age, indication, type and dose of gonadotropins, duration of stimulation.

	Coefficient	95% C.I.	P value
**Pretreatment**	2.4	3.15 to 1.76	<0.001
**Age**	-.28	-.38 to -.18	<0.001
**Indication of infertility**			
**Male factor**	-	-	
**Endometriosis**	-1.59	-4.66 to 1.48	0.31
**Age/ovarian Insufficiency**	0.79	-.67 to 2.26	0.28
**Idiopathic**	3.07	0.29 to 5.85	0.3
**PCOS**	1.48	-.33 to 3.31	0.11
**Tubal**	0.64	-1.56 to 2.86	0.56
**Dose of gonadotropins (IU)**	-.007	-.01 to 0.00	0.06
**Type of gonadotropins**	-.59	-.1.41 to 0.21	0.15
**Duration of stimulation (days)**	-.14	-.34 to 0.05	0.15

Note. C.I. (confidence interval)

## Discussion

The result of this retrospective study indicated that a 3-day course of GnRH antagonist pretreatment increases the number of COCs and MII oocytes obtained after ovarian stimulation compared to conventional antagonist protocol. However, it has to be mentioned that a higher starting dose and consumption of gonadotropins were observed in patients who received GnRH antagonist pretreatment, while stimulation length was equivalent between the two groups. As expected, we noted that patients’ older age had a significant negative impact on the number of retrieved COCs. These findings confirm the results of an older study from our group in which an association between early follicular phase GnRH antagonist pretreatment and a trend toward a higher number of retrieved oocytes was demonstrated in women aged <36 years who underwent fixed GnRH antagonist protocol. However, in spite of the promising findings, caution needed to be applied when interpreting the results, as the study was a small pilot trial [16].

The same topic was recently investigated by a Randomized Controlled Trial (RCT) including 136 normal ovulatory women undergoing IVF/ICSI with r-FSH in a flexible GnRH antagonist protocol. The patients were randomized into two equal groups with or without GnRH antagonist administration from day 2 of the menstrual cycle for 3 days before stimulation. In contrast with our results, this study findings showed that the number of retrieved oocytes did not significantly vary between the two groups. Limitations were underlined by the authors who auspicated for a larger future multicentre trial to confirm their conclusions [22].

Similarly, a case-control study by Viardot-Foucault et al. (2015) reported no difference in terms of number of collected oocytes between 70 patients undergoing GnRH antagonist pretreatment before ovarian stimulation with flexible GnRH antagonist protocol and the control group [23]. In disagreement with our findings, this study results described that in the pretreatment antagonist group a significant lower total dose of rFSH was used for ovarian stimulation compared to the control group. However, these findings were flawed by the semi-retrospective design of the study which represented a potential bias.

Furthermore, the use of GnRH antagonist pretreatment has been also investigated in specific groups of subfertile patients such as poor ovarian responders (PORs). A multicenter RCT including 160 PORs selected according to Bologna Criteria evaluated reproductive outcomes between two equal groups obtained after randomization [24]. Group I received standard ovarian stimulation in a flexible GnRH antagonist protocol, Group II underwent flexible GnRH antagonist protocol preceded by GnRH antagonist pretreatment administered from day 2 to day 8 of the menstrual cycle. Conclusions showed that delayed start protocol significantly improved clinical pregnancy rate (CPR) and IVF cycle parameters in PORs. Indeed, a statistically significant higher number of fertilized and metaphase II oocytes as well as grade I embryos were reported in Group II compared to Group I. Finally, a small RCT by Aflatoonian et al. (2017) compared reproductive outcomes obtained in 60 PORs selected according to Bologna criteria. Patients were randomly assigned to two groups: case group (*n* = 30) in which delayed start GnRH antagonist protocol was initiated from day 2 to 8 of the menstrual cycle immediately after estrogen priming treatment administered from day 21 of the previous cycle for 10 days onwards (double suppression) and control group (*n* = 30) treated with only estrogen priming treatment and antagonist protocol. Results showed no statistically significant difference between the two groups in terms of oocyte maturation and embryo formation rates. On the other hand, a trend toward higher implantation, chemical, clinical and ongoing pregnancy rates was described in delayed start cycles, although it was not statistically significant [25].

A major strength of the presented study relies on its large sample size. Nonetheless, it’s essential to acknowledge certain limitations when interpreting the results, particularly the inherent risk of bias due to the retrospective nature of the study. Despite significant efforts has been made to eliminate all recognized sources of systematic error through multivariable analysis, latent sources of bias may persist. Moreover, it is important to mention that our study population included patients who did not get pregnant after one standard GnRH antagonist stimulation cycle and proceeded with another GnRH antagonist stimulation cycle preceded by early administration of GnRH antagonist pretreatment. This category may represent patients with a potential suboptimal prognosis. In addition, the inclusion of potential PORs among our cohort cannot be excluded, as patients’ age range for inclusion in the study was from 20 to 44 years. Besides, the impact of GnRH antagonist pretreatment on pregnancy rate cannot be assessed because of the study crossover design.

## Conclusions

A 3-day pretreatment course with a GnRH antagonist administered in the early follicular phase seems to increase the number of oocytes retrieved in a GnRH antagonist stimulation protocol. Furthermore, as the initiation of ovarian stimulation in a GnRH antagonist protocol relies on the unpredictable occurrence of spontaneous menses, addition of three days of GnRH antagonist pretreatment may enhance scheduling flexibility without reducing efficacy. Larger cohort studies are needed to validate these findings.

## Supporting information

S1 File Database. (XLSX)

S2 File Database legend. (DOCX)

## Abbreviations

GnRH: Gonadotropin-releasing hormone
ART: Assisted Reproductive Technique
OHSS: Ovarian Hyperstimulation Syndrome
OCP: Oral Contraceptive Pill
OPR: Ongoing Pregnancy Rate
LBRs: Live Birth Rates
rFSH: Recombinant Follicle-Stimulating Hormone
OS: Ovarian Stimulation
OS: Ovarian Stimulation
IVF: In Vitro Fertilisation
ICSI: Intracytoplasmic Sperm Injection
IVM: In vitro Maturation
ET: Embryo Transfer
HRT: Hormonal Replacement Therapy
NC: Natural Cycle
IU: International Units
GEE: Generalized Estimating Equations
ORs: Adjusted Odds Ratios
CIs: Confidence Intervals
PCOS: Polycystic Ovary Syndrome
AMH: Anti-Müllerian Hormone
FSH: Follicle-Stimulating Hormone
E2: 17 b Estradiol
P: Progesterone
LH: Luteinizing Hormone
RCT: Randomized Controlled Trial
PORs: Poor Ovarian Responders
CPR: Clinical Pregnancy Rate

## References

[1] Van Hooren HG, Fischl F, Aboulghar, Marès, Nicollet B, Behre HM, et al. Comparable clinical outcome using the GnRH antagonist ganirelix or a long protocol of the GnRH agonist triptorelin for the prevention of premature LH surges in women undergoing ovarian stimulation. Hum Reprod [Internet]. 2001 [cited 2022 Sep 16];16(4):644–51. Available from: https://pubmed.ncbi.nlm.nih.gov/11278211/ doi: 10.1093/humrep/16.4.644 11278211

[2] Al-Inany HG, Abou-Setta AM, Aboulghar M. Gonadotrophin-releasing hormone antagonists for assisted conception. In: Cochrane Database of Systematic Reviews [Internet]. John Wiley & Sons, Ltd; 2006 [cited 2022 Sep 16]. Available from: https://pubmed.ncbi.nlm.nih.gov/16855976/10.1002/14651858.CD001750.pub2 16855976

[3] Devroey P, Aboulghar M, Garcia-Velasco J, Griesinger G, Humaidan P, Kolibianakis E, et al. Improving the patient’s experience of IVF/ICSI: A proposal for an ovarian stimulation protocol with GnRH antagonist co-treatment [Internet]. Vol. 24, Human Reproduction. Oxford University Press; 2009 [cited 2022 Sep 16]. p. 764–74. Available from: https://pubmed.ncbi.nlm.nih.gov/19153090/19153090 10.1093/humrep/den468

[4] Kolibianakis EM, Collins J, Tarlatzis BC, Devroey P, Diedrich K, Griesinger G. Among patients treated for IVF with gonadotrophins and GnRH analogues, is the probability of live birth dependent on the type of analogue used? A systematic review and meta-analysis [Internet]. Vol. 12, Human Reproduction Update. Hum Reprod Update; 2006 [cited 2022 Sep 16]. p. 651–71. Available from: https://pubmed.ncbi.nlm.nih.gov/16920869/16920869 10.1093/humupd/dml038

[5] Fanchin R, Salomon L, Castelo-Branco A, Olivennes F, Frydman N, Frydman R. Luteal estradiol pre-treatment coordinates follicular growth during controlled ovarian hyperstimulation with GnRH antagonists. Hum Reprod [Internet]. 2003 [cited 2022 Sep 16];18(12):2698–703. Available from: https://pubmed.ncbi.nlm.nih.gov/14645194/ doi: 10.1093/humrep/deg516 14645194

[6] Guivarc’h-Levêque A, Arvis P, Bouchet JL, Broux PL, Moy L, Priou G, et al. Efficacité de la programmation des cycles FIV en antagonistes par les estrogènes. Gynecol Obstet Fertil [Internet]. 2010 [cited 2022 Sep 16];38(1):18–22. Available from: https://pubmed.ncbi.nlm.nih.gov/20022282/20022282 10.1016/j.gyobfe.2009.04.028

[7] Levy MJ, Ledger W, Kolibianakis EM, IJzerman-Boon PC, Gordon K. Is it possible to reduce the incidence of weekend oocyte retrievals in GnRH antagonist protocols. Reprod Biomed Online [Internet]. 2013 [cited 2022 Sep 16];26(1):50–8. Available from: https://pubmed.ncbi.nlm.nih.gov/23177412/ doi: 10.1016/j.rbmo.2012.09.014 23177412

[8] Tremellen KP, Lane M. Avoidance of weekend oocyte retrievals during GnRH antagonist treatment by simple advancement or delay of hCG administration does not adversely affect IVF live birth outcomes. Hum Reprod [Internet]. 2010 [cited 2022 Sep 16];25(5):1219–24. Available from: https://pubmed.ncbi.nlm.nih.gov/20215127/ doi: 10.1093/humrep/deq059 20215127

[9] Griesinger G, Kolibianakis EM, Venetis C, Diedrich K, Tarlatzis B. Oral contraceptive pretreatment significantly reduces ongoing pregnancy likelihood in gonadotropin-releasing hormone antagonist cycles: An updated meta-analysis. Fertil Steril [Internet]. 2010 Nov [cited 2022 Sep 16];94(6):2382–4. Available from: https://pubmed.ncbi.nlm.nih.gov/20537631/ doi: 10.1016/j.fertnstert.2010.04.025 20537631

[10] Farquhar C, Rombauts L, Kremer JAM, Lethaby A, Ayeleke RO. Oral contraceptive pill, progestogen or oestrogen pretreatment for ovarian stimulation protocols for women undergoing assisted reproductive techniques [Internet]. Vol. 2017, Cochrane Database of Systematic Reviews. John Wiley and Sons Ltd; 2017 [cited 2021 Oct 31]. Available from: https://pubmed.ncbi.nlm.nih.gov/28540977/10.1002/14651858.CD006109.pub3 PMC6481489 28540977

[11] Lu Y, Wang Y, Zhang T, Wang G, He Y, Lindheim SR, et al. Effect of pretreatment oral contraceptives on fresh and cumulative live birth in vitro fertilization outcomes in ovulatory women. Fertil Steril [Internet]. 2020 Oct 1 [cited 2022 Oct 7];114(4):779–86. Available from: https://pubmed.ncbi.nlm.nih.gov/32741621/ doi: 10.1016/j.fertnstert.2020.05.021 32741621

[12] Griesinger G, Venetis CA, Marx T, Diedrich K, Tarlatzis BC, Kolibianakis EM. Oral contraceptive pill pretreatment in ovarian stimulation with GnRH antagonists for IVF: a systematic review and meta-analysis. Fertil Steril [Internet]. 2008 Oct [cited 2022 Sep 16];90(4):1055–63. Available from: https://pubmed.ncbi.nlm.nih.gov/18054003/ doi: 10.1016/j.fertnstert.2007.07.1354 18054003

[13] Albano C, Felberbaum RE, Smitz J, Riethmüller-Winzen H, Engel J, Diedrich K, et al. Ovarian stimulation with HMG: Results of a prospective randomized phase III European study comparing the luteinizing hormone-releasing hormone (LHRH)-antagonist cetrorelix and the LHRH-agonist buserelin. Hum Reprod [Internet]. 2000 [cited 2022 Sep 16];15(3):526–31. Available from: https://pubmed.ncbi.nlm.nih.gov/10686191/10686191 10.1093/humrep/15.3.526

[14] Åbyholm T, Barlow D, Devroey P, Diedrich K, Donnez J, Von Düring V, et al. Treatment with the gonadotrophin-releasing hormone antagonist ganirelix in women undergoing ovarian stimulation with recombinant follicle stimulating hormone is effective, safe and convenient: Results of a controlled, randomized, multicentre trial. Hum Reprod [Internet]. 2000 [cited 2021 Nov 8];15(7):1490–8. Available from: https://pubmed.ncbi.nlm.nih.gov/10875855/10875855 10.1093/humrep/15.7.1490

[15] Blockeel C, Baumgarten M, De Vos M, Verheyen G, Devroey P. Administration of GnRH Antagonists in Case of Elevated Progesterone at Initiation of the Cycle: A Prospective Cohort Study. Curr Pharm Biotechnol [Internet]. 2011 Feb 7 [cited 2022 Sep 16];12(3):423–8. Available from: https://pubmed.ncbi.nlm.nih.gov/21133851/ doi: 10.2174/138920111794480633 21133851

[16] Blockeel C, Riva A, De Vos M, Haentjens P, Devroey P. Administration of a gonadotropin-releasing hormone antagonist during the 3 days before the initiation of the in vitro fertilization/intracytoplasmic sperm injection treatment cycle: Impact on ovarian stimulation. A pilot study. Fertil Steril [Internet]. 2011 [cited 2022 Sep 16];95(5). Available from: https://pubmed.ncbi.nlm.nih.gov/21300334/ doi: 10.1016/j.fertnstert.2011.01.028 21300334

[17] Younis JS, Soltsman S, Izhaki I, Radin O, Bar-Ami S, Ben-Ami M. Early and short follicular gonadotropin-releasing hormone antagonist supplementation improves the meiotic status and competence of retrieved oocytes in in vitro fertilization-embryo transfer cycles. Fertil Steril [Internet]. 2010 Sep [cited 2022 Sep 16];94(4):1350–5. Available from: https://pubmed.ncbi.nlm.nih.gov/19800061/ doi: 10.1016/j.fertnstert.2009.08.033 19800061

[18] Popovic-Todorovic B, Racca A, Blockeel C. Added value today of hormonal measurements in ovarian stimulation in gonadotropin-releasing hormone antagonist treatment cycle [Internet]. Vol. 30, Current Opinion in Obstetrics and Gynecology. Lippincott Williams and Wilkins; 2018 [cited 2021 Feb 14]. p. 145–50. Available from: https://pubmed.ncbi.nlm.nih.gov/29664792/29664792 10.1097/GCO.0000000000000459

[19] Humaidan P, Polyzos NP, Alsbjerg B, Erb K, Mikkelsen AL, Elbaek HO, et al. GnRHa trigger and individualized luteal phase hCG support according to ovarian response to stimulation: Two prospective randomized controlled multi-centre studies in IVF patients. Hum Reprod [Internet]. 2013 [cited 2021 Feb 14];28(9):2511–21. Available from: https://pubmed.ncbi.nlm.nih.gov/23753114/ doi: 10.1093/humrep/det249 23753114

[20] Andersen AN, Popovic-Todorovic B, Schmidt KT, Loft A, Lindhard A, Højgaard A, et al. Progesterone supplementation during early gestations after IVF or ICSI has no effect on the delivery rates: A randomized controlled trial. Hum Reprod [Internet]. 2002 [cited 2021 Mar 28];17(2):357–61. Available from: https://pubmed.ncbi.nlm.nih.gov/11821278/ doi: 10.1093/humrep/17.2.357 11821278

[21] Liu XR, Mu HQ, Shi Q, Xiao XQ, Qi HB. The optimal duration of progesterone supplementation in pregnant women after IVF/ICSI: A meta-analysis [Internet]. Vol. 10, Reproductive Biology and Endocrinology. Reprod Biol Endocrinol; 2012 [cited 2021 Mar 28]. Available from: https://pubmed.ncbi.nlm.nih.gov/23237065/10.1186/1477-7827-10-107 PMC3551800 23237065

[22] Zhang Y, Liu L, Qin J, Huang H, Xue L, Wang S, et al. Evaluation of GnRH antagonist pretreatment before ovarian stimulation in a GnRH antagonist protocol in normal ovulatory women undergoing IVF/ICSI: a randomized controlled trial. Reprod Biol Endocrinol [Internet]. 2021 Dec 1 [cited 2022 Sep 16];19(1). Available from: https://pubmed.ncbi.nlm.nih.gov/34641897/10.1186/s12958-021-00836-8 PMC8507211 34641897

[23] Viardot-Foucault V, Nadarajah S, Lye WK, Tan HH. GnRH antagonist pre-treatment: One centre’s experience for IVF-ICSI cycle scheduling. Reprod Biomed Online. 2015 Apr 1;30(4):366–72. doi: 10.1016/j.rbmo.2014.11.018 25684093

[24] Maged AM, Nada AM, Abohamila F, Hashem AT, Mostafa WAI, Elzayat AR. Delayed Start Versus Conventional GnRH Antagonist Protocol in Poor Responders Pretreated with Estradiol in Luteal Phase: A Randomized Controlled Trial. Reprod Sci [Internet]. 2015 Dec 1 [cited 2022 Sep 16];22(12):1627–31. Available from: https://pubmed.ncbi.nlm.nih.gov/26045549/ doi: 10.1177/1933719115590666 26045549

[25] Aflatoonian A, Hosseinisadat R, Baradaran R, Mojtahedi MF. Pregnancy outcome of “delayed start” GnRH antagonist protocol versus GnRH antagonist protocol in poor responders: A clinical trial study. Int J Reprod Biomed [Internet]. 2017 Apr 1 [cited 2022 Sep 16];15(4):231–8. Available from: https://pubmed.ncbi.nlm.nih.gov/28835940/ doi: 10.29252/ijrm.15.4.231 28835940 PMC5555041

