# Correction: Impact of GnRH antagonist pretreatment on oocyte yield after ovarian stimulation: A retrospective analysis

**DOI:** 10.1371/journal.pone.0324687

**Published:** 2025-05-22

**Authors:** The PLOS One Staff

## Notice of Republication

This article was republished on April 25, 2025, to correct an error in the author list: Federica Di Guardo was erroneously displayed as Di Guardo Federica.

There is an error in affiliation 4 for authors Panagiotis Drakopoulos. The correct affiliation 4 is: Institute of life, IVF clinic, Athens, Greece

An additional affiliation is missing for the fourth author. Panagiotis Drakopoulos is also affiliated with the School of Medicine, European University Cyprus, Engomi, Nicosia, Cyprus.

The publisher apologises for the error. Please download this article again to view the correct version. The originally published, uncorrected article and the republished, corrected article are provided here for reference.

**Supporting Information**

**S1 File.** Originally published, uncorrected article.

**S2 File.** Republished, corrected article.
