# Supplementary material for: Impact of GnRH antagonist pretreatment on oocyte yield after ovarian stimulation: A retrospective analysis
Source: PLoS One. 2024 Oct 7;19(10):e0308666. doi: 10.1371/journal.pone.0308666 (PMC11458021; doi:10.1371/journal.pone.0308666)
Supplement: S2 File — (DOCX) [file pone.0308666.s002.docx]

|  | Pretreatment (D=0) | No Pretreatment (D=1) |  |
| --- | --- | --- | --- |
| **Demographics** |  |  |  |
| indication |  |  | N |
| age |  |  | R |
| AMH |  |  | J |
| **Endocrinology** |  |  |  |
| Progesterone basal |  |  | B |
| E2 basal |  |  | A |
| LH basal |  |  | C |
| FSH basal |  |  | D |
| Progesterone at trigger |  |  | F |
| E2 at trigger |  |  | E |
| LH at trigger |  |  | G |
| FSH at trigger |  |  | H |
| **Stimulation characteristics** |  |  |  |
| Starting dose of gonadotropin (IU) |  |  | M |
| Total consumption of gonadotrophins (IU) |  |  | Q |
| Duration (days) of ovarian stimulation |  |  | P |
| Type of gonadotropin |  |  | L |
| **Embryology** |  |  |  |
| COC |  |  | S |
| MII |  |  | T |
| Fertilization rate |  |  | AA |
| number of frozen embryos d3 |  |  | AF |
| Number of frozen embryos d5/6 |  |  | AK |
| embryo utilization rate (defined as the number of embryos utilized (transferred or cryopreserved) per number of 2PN zygotes) |  |  | AM |
| Cycle outcome |  |  | I |
| Incidence and severity of OHSS |  |  | K |

**EXCEL FILE PRETREATMENT LEGEND**
